# Bioluminescent magnetic nanoparticles as potential imaging agents for mammalian spermatozoa

**DOI:** 10.1186/s12951-016-0168-y

**Published:** 2016-03-17

**Authors:** Erick S. Vasquez, Jean M. Feugang, Scott T. Willard, Peter L. Ryan, Keisha B. Walters

**Affiliations:** Department of Chemical and Materials Engineering, University of Dayton, Dayton, OH 45469 USA; Facility for Cellular and Organismal Imaging, Mississippi State University, Mississippi State, MS 39762 USA; Department of Animal and Dairy Sciences, Mississippi State University, Mississippi State, MS 39762 USA; Department of Biochemistry and Molecular Biology, Entomology and Plant Pathology, Mississippi State University, Mississippi State, MS 39762 USA; Department of Pathology and Population Medicine, College of Veterinary and Medicine, Mississippi State University, Mississippi State, MS 39762 USA; Dave C. Swalm School of Chemical Engineering, Mississippi State University, Mississippi State, MS 39762 USA

**Keywords:** Spermatozoa, Bioluminescence Imaging, Magnetic nanoparticles, Nanocomposites, Reproduction, Core–shell nanoparticles, Luciferase

## Abstract

**Background:**

Nanoparticles have emerged as key materials for developing applications in nanomedicine, nanobiotechnology, bioimaging and theranostics. Existing bioimaging technologies include bioluminescent resonance energy transfer-conjugated quantum dots (BRET-QDs). Despite the current use of BRET-QDs for bioimaging, there are strong concerns about QD nanocomposites containing cadmium which exhibits potential cellular toxicity.

**Results:**

In this study, bioluminescent composites comprised of magnetic nanoparticles and firefly luciferase (*Photinus pyralis*) are examined as potential light-emitting agents for imaging, detection, and tracking mammalian spermatozoa. Characterization was carried out using infrared spectroscopy, TEM and cryo-TEM imaging, and ζ-potential measurements to demonstrate the successful preparation of these nanocomposites. Binding interactions between the synthesized nanoparticles and spermatozoon were characterized using confocal and atomic/magnetic force microscopy. Bioluminescence imaging and UV–visible-NIR microscopy results showed light emission from sperm samples incubated with the firefly luciferase-modified nanoparticles. Therefore, these newly synthesized luciferase-modified magnetic nanoparticles show promise as substitutes for QD labeling, and can potentially also be used for in vivo manipulation and tracking, as well as MRI techniques.

**Conclusions:**

These preliminary data indicate that luciferase-magnetic nanoparticle composites can potentially be used for spermatozoa detection and imaging. Their magnetic properties add additional functionality to allow for manipulation, sorting, or tracking of cells using magnetic techniques.

**Electronic supplementary material:**

The online version of this article (doi:10.1186/s12951-016-0168-y) contains supplementary material, which is available to authorized users.

## Background

Nanoparticle-based biomedical applications include nanomedicine, bioimaging and theranostics [1]. Recently, nanoparticle-based bioimaging technologies have focused on bioluminescent resonance energy transfer-conjugated quantum dots (BRET-QDs), such as PbS, CdSe/ZnS, and CdTe/CdS QDs [2–6]. However, BRET-QDs are under scrutiny due to their cadmium content, since cadmium has known toxicity [7, 8]. An alternative noninvasive bioimaging system could explore luciferase enzymes, which are found in nature and has inherent light emission characteristics, for bioluminescence imaging in whole animal and cellular systems. For example, luciferase obtained from *Renilla reniformis* has been coupled with CdSe/ZnS QDs to create self-illuminating nanoparticles for dual imaging purposes. In this complex, the chemical energy generated by the reaction of luciferase with its substrate (coelenterazine or luciferin) produced light (bioluminescence) which excited the QD for a bright fluorescence emission [9, 10]. Similarly, firefly *Photinus pyralis* luciferase has been combined with core–shell quantum rods (CdSe/CdS or CdSe/CdS/ZnS), producing a significant increase and optimization in BRET ratios [11].

In biomedical applications, core–shell nanostructures comprised of a magnetic core present a unique opportunity for multi-functionality, incorporating optical imaging with tracking, sorting and/or cellular manipulation [12, 13]. Superparamagnetic iron oxide nanoparticles are clinically approved by the European Medicines Agency (EMA) and U. S. Food and Drug Administration (FDA), and have been used to label and track cells via MRI techniques [14]. For example, lectin-coated iron oxide nanoparticles have been successfully used to bind and remove (under magnetic field) moribund mammalian spermatozoa without impairing the fertility potential of remaining unbound spermatozoa [15, 16]. Recently, hybrid micro-helixes made of a polymer-metal composite with magnetic properties have demonstrated the possible impacts from using magnetic microstructures in assisted fertilization [17]. Magnetic nanoparticles have shown viability in labeling and tracking applications; however, we are interested in using magnetic nanoparticles to enable cell detection, labeling and sorting without further perturbation of their viability—which would be the case when using fluorescent agents requiring additional excitation. In this study, coupling firefly luciferase (*Photinus pyralis)* with a magnetic nanoparticle carrier is expected to provide a multifunctional nanocomposite with both magnetic manipulation and bioimaging properties. One objective of this work is to describe the synthesis and in situ characterization of core–shell nanocomposites comprised of a citric acid-stabilized magnetic nanoparticle core surrounded by a spherical shell of the bioluminescent firefly luciferase (*Photinus pyralis*) enzyme. A second objective is to analyze preliminary bioluminescence data from boar spermatozoa incubated with the newly synthesized luciferase-modified magnetic nanoparticles (Luc + MNP). This analysis is presented using chemical and morphological characterization of the luciferase-magnetic nanoparticle composite and bioluminescence imaging, along with comparative data from a commercial BRET-QD that served as bioluminescence control.

## Results and discussion

### Conjugated nanoparticle preparation

Iron oxide magnetic nanoparticles (MNPs) were synthesized using a co-precipitation technique that is described in detail in the methods section. After synthesis, MNPs were stabilized with a citric acid coating to form the citric acid-magnetic nanoparticle (CA-MNP) conjugate. A second reaction added the firefly luciferase enzyme onto the periphery of the CA-MNP to form the luciferase-CA-MNP (Luc + MNP) complex. With the goal of performing cell sorting through nanotechnology tools, one advantage of using luciferase enzymes as imaging probes resides on the avoidance of additional light excitation that may damage cells when using fluorescent probes [18].

**Fig. 1:**
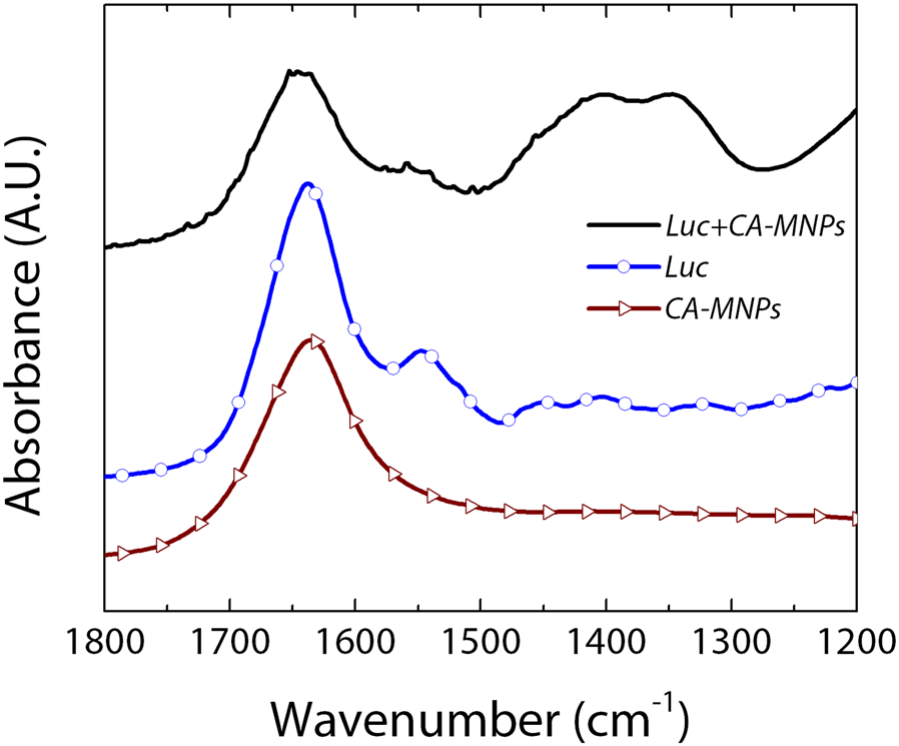
FTIR spectra for **a** citric acid-coated magnetic nanoparticles (CA-MNPs), **b** firefly luciferase (Luc), and **c** firefly luciferase combined with CA-MNP (Luc + MNPs) confirm the successful surface modification steps

*In situ* ATR-FTIR spectroscopy was used to confirm the chemical changes in the MNPs for each reaction step (Fig. 1). For CA-MNPs, a strong peak at ~1645 cm^−1^ was observed corresponding to the symmetric carbonyl (C = O) vibrations of the carboxylic acid groups (–COOH) in citric acid when bound to iron oxide [19]. FTIR spectra of neat firefly luciferase showed distinctive peaks at 1550 cm^−1^ and 1515 cm^−1^, which are amide-II vibrations characteristic of luciferase [20]. In addition, the peak near 1650 cm^−1^ corresponds to an amide-I band commonly observed when multiple α- and β-functional groups are present, and has been previously observed for luciferase [21]. After addition of luciferase to the CA-MNPs complex, distinctive peaks were observed for the Luc + MNP samples that match the spectral signature for neat luciferase and indicate strong binding between luciferase and the CA-MNPs. Amide-II peaks observed in the neat luciferase spectra were also observed in the luciferase-CA-MNP (Luc + MNP) spectra. In addition, the strong peaks at ~1400 and ~1350 cm^−1^ and the broadening and slight shift of the Amide-I peak is indicative of strong binding interactions between the amide/amine groups of luciferase and the carboxylic acid groups on the surface of the citric-acid modified iron oxide nanoparticles [19].

**Fig. 2:**
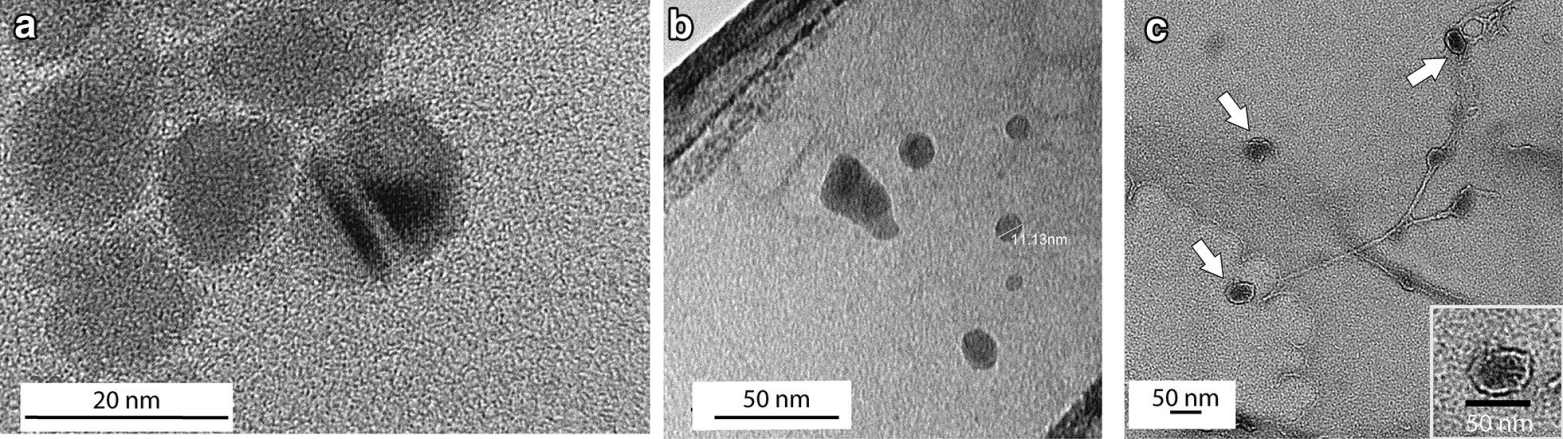
Citric acid-modified magnetic nanoparticles (CA-MNPs) were observed **a** with TEM to be ~17 nm in diameter and **b** with cryo-TEM to be similarly sized but dispersed. After the addition of the luciferase, **c** cryo-TEM shows that the Luc + MNP are larger (~ 40–50 nm) with a core–shell structure where a lighter luciferase shell surrounds the darker CA-MNP core; the *inset* shows a single magnified core–shell Luc + MNP structure showing the luciferase shell thickness is ~5 nm

Transmission electron microscopy (TEM) was used to examine the structure and uniformity of the synthesized nanocomposites. Figure 2a shows a high-resolution TEM image of the as-synthesized CA-MNPs; homogeneous particles, approximately 17 nm in diameter, were observed. Cryo-TEM was performed on CA-MNPs to confirm the primary particle size and gain information on the in situ nanoparticle dispersion (Fig. 2b). Samples imaged under cryogenic conditions showed a more dispersed particle phase that is expected to be more representative of the actual dispersion in solution [22]. Cryo-TEM images of Luc + MNPs (Fig. 2c) show the nanocomposite diameter ranging from 40–50 nm were observed, supporting the addition of firefly luciferase to the nanoparticles. Luc + MNP showed as distinct core–shell morphology with a lighter- colored luciferase shell (~5 nm) surrounding the darker CA-MNP core. (Note that the lighter-colored strands between and near some of the Luc + MNP structures are likely luciferase strands which have extended/unfolded during the cryogenic sample preparation and are partially coordinated with the Luc + MNPs as an effect of rapid temperature changes as discussed elsewhere [23]). These TEM images not only validate the formation of the Luc + MNP complexes, but also demonstrate the formation of core-shell structures, as has been reported elsewhere for luciferase complexation with silver nanoparticles [24].

Dynamic light scattering (DLS) was used to evaluate the diameter of CA-MNP both before (31.5 ± 1.5 nm) and after (119.7 ± 23.9 nm) firefly luciferase adsorption. The increase in particle diameter and particle size distribution (Additional file 1: Figures S1, S2) is attributed to the addition of a firefly luciferase shell. While the trends are the same, the DLS particle sizes are larger than those measured using TEM. However, the DLS data is expected to be more representative of the in situ particle sizes since the DLS data is collected in the luciferase enzyme hydrated state and the DLS experiment gives statistically significant data for the average particle sizes and particle size distributions.

Electrostatic charge on nanoparticle surfaces can be used to confirm the surface modification of nanoparticles, including binding with enzymes and proteins [24]. Here, phase-angle light scattering (PALS) measurements were used to study the surface charge of CA-MNPs at neutral pH before and after the addition of firefly luciferase on the CA-MNP periphery. For CA-MNPs, PALS data shows a negative ζ-potential (−21.5 ± 2.0 mV). After addition of luciferase, the ζ-potential of nanoparticles increased to 4.5 ± 0.5 mV. The shift from negative to positive ζ-potential values confirms that luciferase is present as a shell on the exterior in the Luc + MNPs complexes.

### Spermatozoa labeling and bioluminescence imaging

**Fig. 3:**
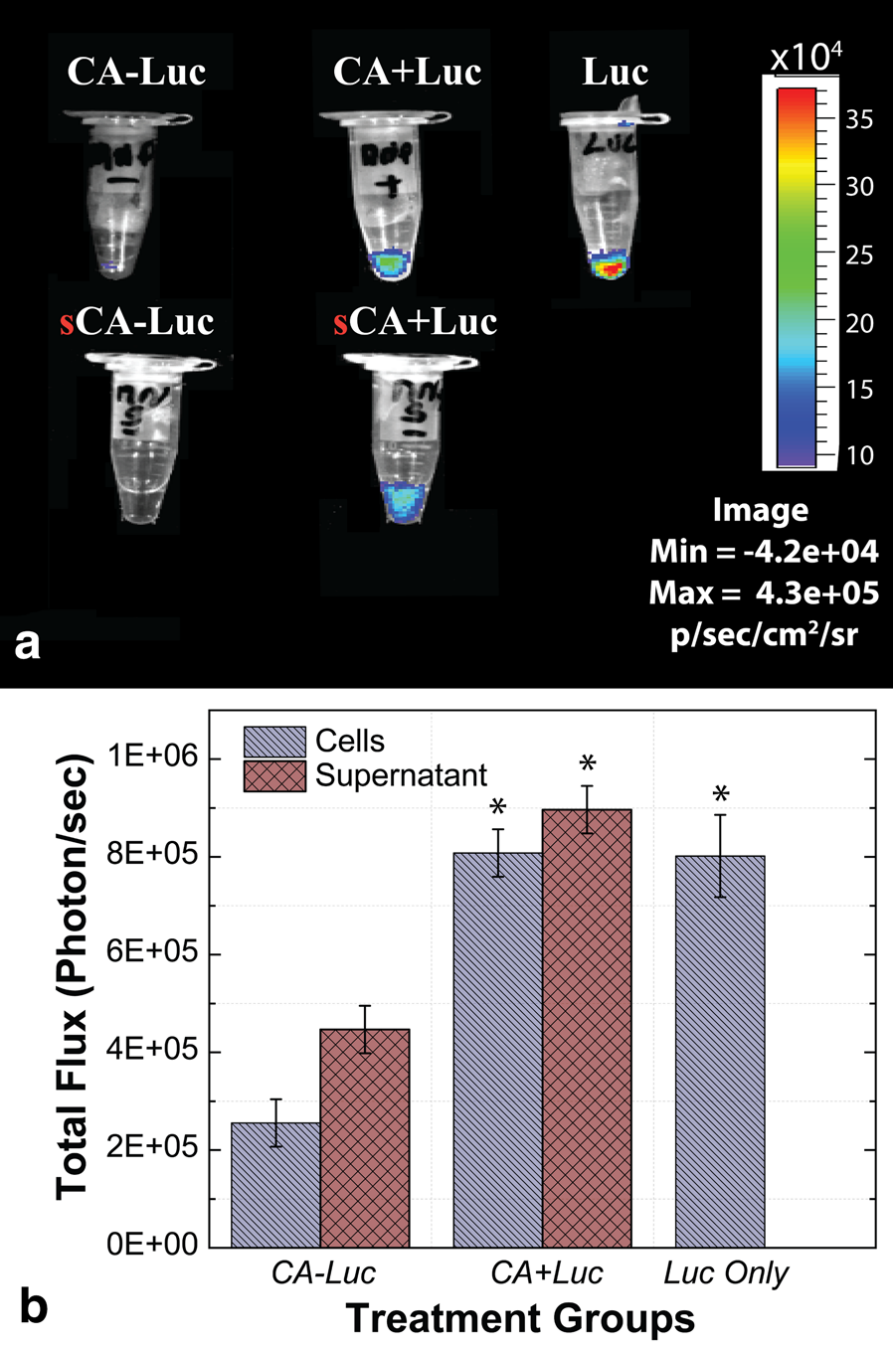
Bioluminescence imaging (BLI) of CA-MNP, Luc + MNPs, and Luc only. Spermatozoa were labeled in PBS solution and then centrifuged. Sperm pellets from the Luc + MNPs, and Luc samples, and their corresponding supernatants (sCA-MNP, sLuc + MNP), were separately imaged after supplementation with coelenterazine. (Note that measurements were performed on 1.5 mL centrifuge tubes with ~50 µL of sample.) **a** Sample images with light (BLI) signal intensities shown as a *gradient color scale*. **b** Quantified BLI signals (mean ± sem). *Asterisk* indicates significant difference from CA-Luc Control (ANOVA-2, P < 0.05, N = 4 replicates)

After confirming the chemical and morphological characteristics of Luc + MNPs using a variety of in situ analyses, the newly synthesized Luc + MNP nanoparticles were evaluated for sperm labeling and imaging with the aid of in situ bioluminescence imaging (BLI) experiments to demonstrate the bioluminescent properties. Labeling procedures and imaging were followed as previously described [2, 15]. In this study, purified motile boar spermatozoa were prepared and labeled without or with the CA-MNP and Luc + MNP nanoparticles. Unlabeled sperm samples and samples labeled with BRET-QD were used as negative and positive controls, respectively. Briefly, PBS-suspended spermatozoa were labeled and then washed three times by centrifugation to remove excess nanoparticles. All sperm pellets and their corresponding supernatants were mixed with luciferase substrate (coelenterazine) and immediately imaged (In Vivo Imaging System, IVIS 100; Xenogen; Fig. 3). Compared to CA-MNP and supernatant, BLI signal intensity was higher in spermatozoa samples incubated with Luc + MNPs. For the Luc + MNP sample, relative BLI intensities for the sperm pellet and supernatant indicate that while excess unbound MNPs are present in the supernatant, there is a higher signal from the sperm pellet and indicates positive interactions between the spermatozoa and Luc + MNP. Significantly lower BLI intensities were observed for the CA-MNP sample and supernatant as compared with neat luciferase and Luc + MNP samples (Fig. 3). Therefore, the strong BLI signal from the Luc + MNP coupled with spermatozoa, before and after centrifugation, confirms the presence and viability of the MNP- bound luciferase (Luc + MNP) as an image agent. The BLI for neat luciferase (with coelenterazine added) is presented as a positive control, and as expected shows the highest BLI signal (Luc, Fig. 3a), at least in part because the luciferase is not split between sample and supernatant aliquots. Spermatozoa in the Luc + MNP sample showed slightly lower BLI levels as compared to the luciferase alone, which is certainly due to substantial levels of Luc + MNP in the supernatant.

**Fig. 4:**
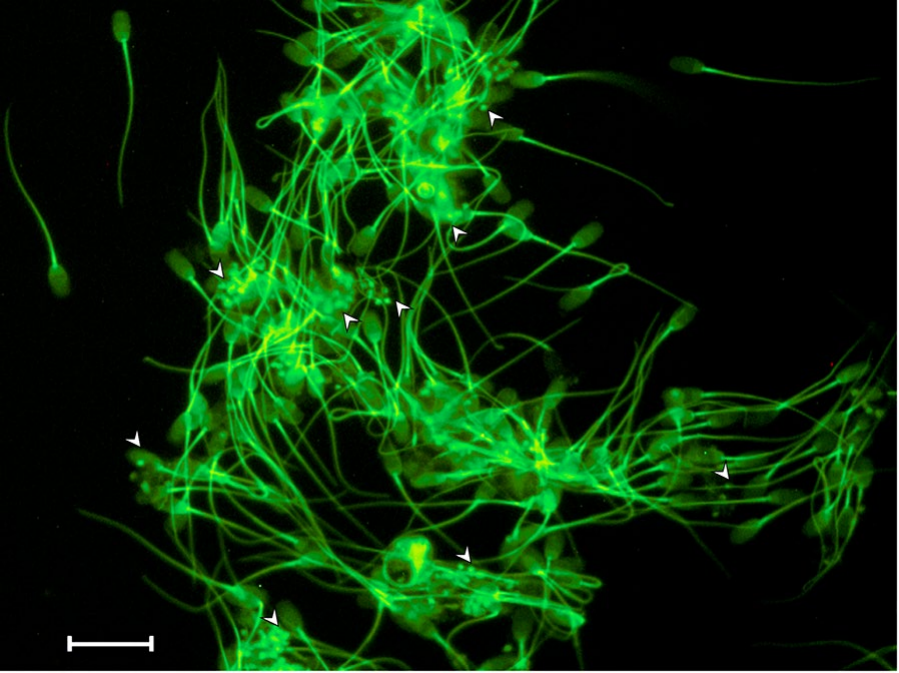
Micrograph of mammalian spermatozoa cells incubated with Luc + MNPs. *Arrow heads* indicate Luc + MNP locations and show the nanoparticles in close proximity to the spermatozoa, with multiple nanoparticles attached to head and/or tail of the sperm cells. *Scale bar* 10 microns

After in situ bioluminescence imaging, the sperm Luc + MNP labeled samples were smeared on microscope slides and light emissions imaged using two microscopic techniques, UV–visible-NIR and laser confocal. Using a series of in situ experiments, it was observed that the Luc + MNP and CA-MNP nanoparticles were in a non-aggregated state. It should be noted that the presence of luciferase, in particular while in the presence of biological media such as PBS (which was the as- received solution for boar spermatozoa specimens), could cause aggregation in situ and during drying [25–28]. To examine these issues, we collected an additional TEM image under non-cryo conditions (i.e., evaporative drying) that revealed possible aggregation of Luc + MNPs which could be a result of the PBS solution and/or the drying process (Additional file 1: Figure S3). Despite this observation, we can still investigate one of the primary objectives of this study—a preliminary evaluation of binding interactions between Luc + MNPs and boar spermatozoa. After Luc + MNPs are added to boar sperm, round nanoparticle structures were observed on the surface of the sperm cells using the UV–vis-NIR microscope (see arrow heads, Fig. 4). Control experiments for QDs (Additional file 1: Figure S4) and neat spermatozoa cells (Additional file 1: Figure S5) were conducted, which reveal that the round nanoparticle structure are the MNP. While these preliminary finding confirm that the nanoparticles interact with the cell surface (Fig. 4), the MNP density per cell, cell and MNP agglomeration, and time-dependency of the cell-MNP binding will be examined in future work.

**Fig. 5:**
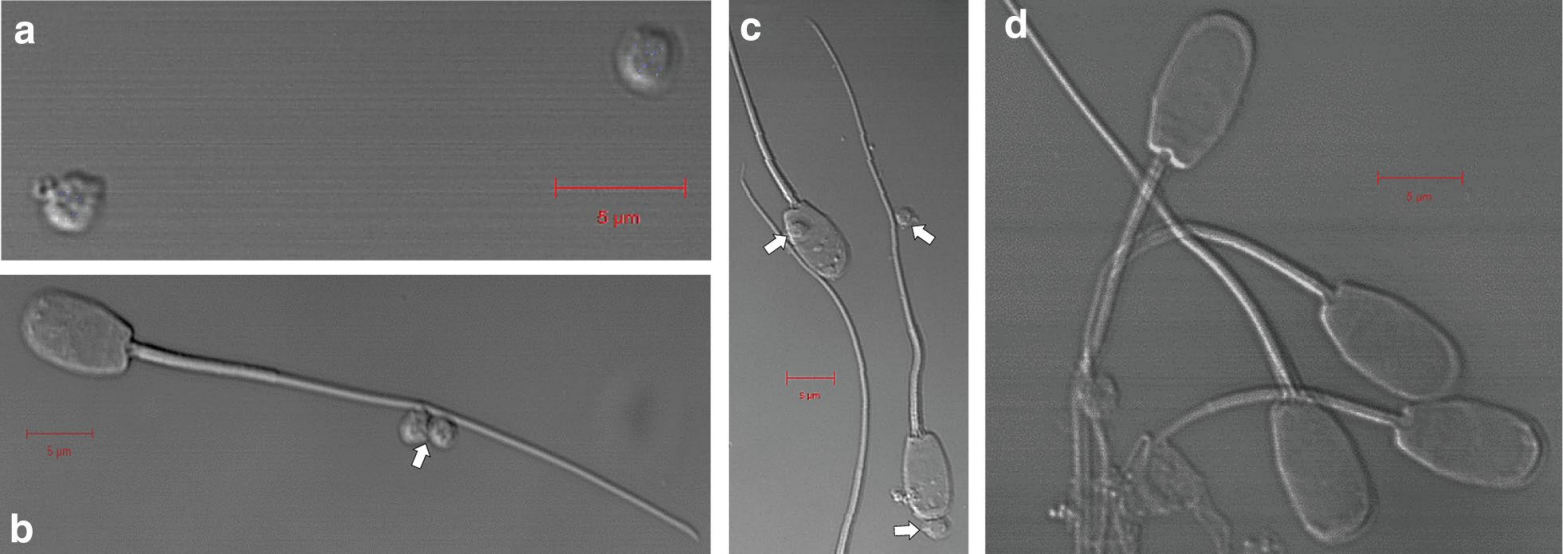
**a** Magnetic nanoparticles (MNPs) modified with luciferase (Luc + MNP) were observed as 1.5–2 µm aggregates under PBS and dried sample conditions. **b** MNP-CA and **c** Luc + MNP bound at different locations (*head* and *tail*) onto spermatozoa. **d** Spermatozoa incubated in PBS show no particles or aggregates. *Arrows* indicate MNP locations and *scale bars* correspond to 5 µm

To gain a better understanding of the nanoparticle interactions with spermatozoa, laser confocal microscopy measurements were performed (Fig. 5). Preliminary analyses on the as-synthesized Luc + MNPs were used as a control experiment to observe any aggregated structures in the presence of PBS (Fig. 5a); some large-scale particle aggregates, ranging from 1.5–2 microns in diameter, were observed. This confirms the TEM results showing that, unlike the cryo-TEM or in situ characterization techniques, aggregation can be caused by PBS and/or the drying conditions used for collecting non-cryo TEM images. Within the aggregated structures, the core–shell structure of the nanoparticles was observed, showing that the luciferase shell is still present on the surface of the MNPs.

**Fig. 6:**
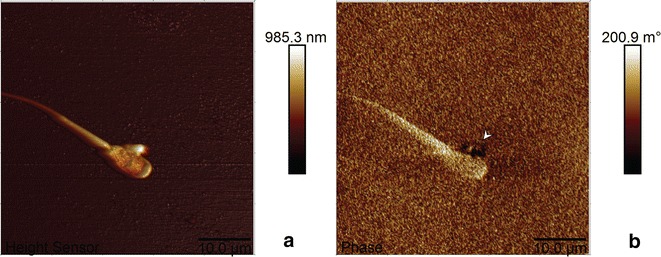
Atomic force and magnetic force microscopy (AFM/MFM) reveals **a** adsorption of Luc + MNP on a spermatozoon head (AFM *height image*) and **b** the magnetic properties of the adsorbed Luc + MNP (*arrow head*) (MFM *lift-phase signal image*)

To obtain some preliminary information on the location and number of interactions between Luc + MNPs and a spermatozoon and on the effect of the shell composition (Luc or CA) on spermatozoa labeling, two separate experiments are discussed. A representative image showing interactions between CA-MNPs and a spermatozoon revealed that the CA-coated particles bind with the tail of the spermatozoon (Fig. 5b). Experiments conducted on Luc + MNPs showed that the particles bind at multiple positions, near the head and/or the tail, of the spermatozoon (Fig. 5c). While these results preliminarily indicate that there may be different interactions between the surface-modified MNPs and spermatozoa based on the exterior (shell) functionality (CA or Luc), the location dependency and density of spermatozoon binding based on different surface modifiers should be extensively investigated in future efforts. A control experiment was performed, and no small or large particles were observed (Fig. 5d). Adding to this control experiment, the magnetic nature of the attached particles was confirmed using a third microscopy technique, atomic and magnetic force microscopy (AFM/MFM), which showed magnetic domains inside the nanoparticle structure (Fig. 6) [27]. The AFM/MFM results demonstrate that the Luc + MNPs retain their magnetic properties even after they are attached to a spermatozoon (Fig. 6b).

This study demonstrates that luciferase-magnetic nanoparticle (Luc + MNP) composites and citric acid-magnetic nanoparticles (CA-MNPs) can be used for binding and imaging of spermatozoa. Luc + MNPs adds additional advantages as different locations on the sperm are targeted for binding and enhanced bioluminescence imaging and tracking can be performed. At this point, their additional magnetic functionality, for example to manipulate the sperm cells, was not examined; however, it will be the focus of future studies. This preliminary study shows that luciferase-modified magnetic nanoparticles is a magnetic platform that can be utilized as a possible alternative for QD-based bioimaging, and which also has potential for magnetic cellular manipulation and MRI applications. In addition, this nanocomposite system will allow for alternate or secondary surface functionality to be included, as desired, through tailored surface-modification techniques. Future studies could include examinations of the (1) optimum ratio(s) between luciferase and CA-MNPs, (2) adsorption process mechanism(s) and the resultant Luc + MNP complex structures using experimental and computational techniques [29], Luc + MNP/cell binding ratios using in vivo*/*vitro microscopy techniques [30], toxicity and biocompatibility effects on the as-synthesized Luc + MNPs [31], and alternate nanoscale fluorescent dyes which are emerging in cell labeling [32].

## Conclusion

Magnetic nanoparticles were synthesized and complexed with firefly (*Photinus pyralis*) luciferase enzyme to produce a multifunctional nanocomposite, Luc + MNP. Inherent bioluminescence in the presence of mammalian spermatozoa was examined, showing Luc + MNP as a promising candidate to enhance or replace some current bioimaging technologies. By utilizing FDA-approved iron oxide magnetic nanoparticles and a natural enzyme, such as firefly luciferase, this nanocomposite has potential for a lower toxicity than quantum dots, as well as the ability to magnetically manipulate cells and track them in vivo. The results presented here demonstrate the possibility of using this luciferase-modified magnetic nanoparticle for cellular binding and imaging. Additional studies on the optimum concentrations and solution composition for cell binding and viability, cell tracking and magnetic manipulation, and time-dependence of the labeling and bioluminescence will allow for a better understanding of the parameters for implementing Luc + MNPs for assisted reproductive technologies. Other activation mechanisms (beyond coelenterazine addition), such as ATP/Mg^2+^ sources, are also interesting avenues for future studies.

## Methods

### Materials

Iron (II) chloride (Sigma Aldrich, 98 %), iron (III) chloride (Alfa Aesar, 98 %), ammonium hydroxide (Acros, 28–30 %), and luciferase from *Photinus pyralis* (firefly) were used as received (Sigma Aldrich, 98 %). Ultrapure type I water (EMD Millipore^®^) was utilized in the preparation of all experimental solutions.

### Luciferase-CA-MNPs synthesis

In brief, iron oxide magnetic nanoparticles (MNPs) were synthesized by combining 10.014 g of iron chloride (II) and 2.665 g of iron chloride (III) in the presence of 0.7 M ammonium hydroxide (high pH) at 60 °C under an inert atmosphere with the aid of mechanical stirring for a final volume of 250 mL. After 30 min of reaction, iron oxide magnetic nanoparticles were separated with an external magnet and washed twice with deionized water. The separated particles were stabilized using a 0.02 g/mL citric acid (CA) solution at 90 °C for 1 h [33]. CA-stabilized magnetic nanoparticles (CA-MNP) were then separated and washed with water twice using an external magnet. CA-MNPs were then added to 100 mL of water and centrifuged at 14,500 rpm until a black precipitate was formed. For Luc adsorption onto CA-MNP, ~10 mg/L CA-MNP was co-incubated with the luciferase (Luc) enzyme (200 mg/L) at ambient temperature to avoid enzyme deactivation. The resultant Luc + MNP complexes, and the CA-MNP samples, were characterized using dynamic light scattering (DLS), Fourier transform infrared (FTIR) spectroscopy, transmission electron microscopy (TEM), and zeta potential measurements. Both the Luc + MNP and CA-MNP control samples were assessed using bioluminescence imaging (BLI) in the presence of spermatozoa.

### Magnetic nanoparticle characterization techniques

#### Attenuated total reflectance-Fourier transform infrared spectroscopy (ATR-FTIR)

A 6700 Nicolet FTIR spectrophotometer from Thermo Electron Corporation with a He–Ne laser MCT-A* detector was used for all measurements. To collect ATR-FTIR spectra, a Miracle-ATR™ external accessory with a diamond-ZnSe crystal (PIKE Technologies) was used for data collection.

#### Transmission electron microscopy (TEM)

A JEOL 2100 operated at 200 kV was used for high- resolution imaging (Additional file 1: Figure S3). Cryo-TEM images were collected with a JEOL 1400 Biological TEM using a Gatan, Inc. Cryoplunge™ adapter (Fig. 2). For all non-cryo TEM imaging, a drop of the liquid samples was deposited on a carbon Formvar^®^ Cu 300 mesh grid.

#### Dynamic light scattering (DLS) and zeta potential measurements

A ZetaPALS instrument (Brookhaven Instruments Corporation, Holtsville, NY) was used for dynamic light scattering (DLS) and zeta potential measurements. A minimum of 5 measurements were collected for each sample.

### Spermatozoa preparation and labeling

Motile boar spermatozoa were selected from freshly collected samples (Prestage Farms, West Point, MS, USA) as described previously [34]. The spermatozoa (2 × 10^8^ sperm/mL) were incubated with 20 μL of CA-MNP and Luc + MNP solution stocks as described in 4.2. The positive and negative controls consisted of spermatozoa incubated with a 1 nM quantum dot (QD; Additional file 1: Figure S4) solution and with phosphate buffered saline (PBS; Additional file 1: Figure S5), respectively.

For the positive control study, a stock solution of CdSe/ZnS core–shell structure QD (500 nM in Tris buffer) cross-linked to *Renilla* luciferase (BRET) and nona-arginine R9 peptide was purchased from Zymera Inc. (San Jose, CA, USA). The BRET-QD complex is a self-illuminating nanoparticle that emits light under incubation with coelenterazine (luciferase substrate) and exhibits intense fluorescence with red-shifted emission (655 nm) following excitation (Additional file 1: Figure S4). Mixtures were incubated at 37 °C for 30 min, and then washed three times with PBS using centrifugation (1000*g*, 3 min). Supernatants containing excess QD were removed and 50 μL of each was retained for BLI. In parallel, sperm pellets were suspended with 50 μL PBS for experiments.

### Confocal, fluorescence, and atomic/magnetic-force microscopy imaging

Aliquots of labeled spermatozoa incubated with only PBS (negative control), CA-MNP in PBS, and Luc + MNP in PBS were placed onto microscope slides to evaluate their optical and fluorescence emission using a Laser confocal microscope (LSM510; Carl Zeiss) and a Nikon Eclipse Ni fluorescence microscope, respectively. For epifluorescence microscopy, aliquots of spermatozoa labeled with QD were also prepared (positive control; Additional file 1: Figure S4), as well as neat spermatozoa (Additional file 1: Figure S5). Images were taken with a Nikon CFI Plan Fluor Ph2 DLL 40× objective Pan Fluor 40×, Ph2 DLL. A Nikon B-2E/C fluorescence filter (96311, green: 465–495 nm excitation and 515–555 nm emission) and Semrock BrightLine^®^ QD655-C single-band filter (395–460 nm excitation and 640–680 nm emission) was used to observe the Luc + MNP and QD nanoparticles, respectively. For AFM/MFM analyses, a drop of Luc + MNPs combined with spermatozoa was placed onto a glass slide and allowed to air dry. A Dimension Icon^®^ atomic force microscope with a Bruker^®^ MESP^®^ tip, under magnetic force microscopy mode, was utilized for AFM/MFM magnetic analysis with a minimum lift height of 200 nm (Fig. 6).

### Bioluminescence imaging (BLI) analysis

BLI analysis was performed as previously reported [2]. Briefly, 4 μg of coelenterazine was added to each of the cell suspensions and supernatants, and gently mixed. All samples were imaged within 10 min using an IVIS 100 Bioluminescence Imaging System (Caliper Life Sciences, Hopkinton, MA, USA) with a 1 min acquisition time and no filter. All images were analyzed using Living Image Software (v2.50, Caliper Life Sciences). Measurements were made by drawing a primary region of interest (ROI) on the bioluminescence images in each sample tube. A secondary ROI surface without sample tubes was measured as the background, and this value subtracted from the primary ROI light emission to correct for auto-fluorescence. The bioluminescence data are presented as total light emission (photons per second).

## Additional file

10.1186/s12951-016-0168-y Hydrodynamic diameter for in-house synthesized CA-MNPs, as measured by DLS number intensity (5 replicates). An average diameter of 31.5 ± 1.5 nm was measured for the in-house synthesized CA-MNPs; **Figure S2.** Hydrodynamic diameter for Luc + MNP, as measured by DLS number intensity (5 replicates). After luciferase addition, the number intensity particle diameter measured increased as compared to CA-MNPs. The larger particle diameter matches the size increases observed by TEM, and confirms that luciferase is bound onto the CA-MNP. Future studies will examine the optimum ratio between luciferase and CA-MNPs, the mechanisms of the adsorption process(es), and the resultant Luc + MNP complex structures. **Figure S3.** Magnetic nanoparticles (Luc + MNPs) aggregated under the presence of PBS solutions and in a conventional TEM characterization where like in the confocal, epifluorescence, and atomic/magnetic force microscopy results dried samples were used for imaging Luc + MNPs interacting with spermatozoa with the drying process contributing to the aggregation observed. **Figure S4.** QDs nanoparticles combined with spermatozoa showed binding at different sites on the cells (*arrows*) as well as unlabeled cells (*arrow heads*). *Scale bar* 10 micrometers. The QD associated with spermatozoa are attached at multiple and different sites on the cell; examples are marked with *arrows*. Note that not allspermatozoa were labeled using the QD method; examples of these unlabeled cells are marked with *arrow heads*. **Figure S5.** Neat spermatozoa observed with an epifluorescence microscope. *Scale bar* 10 microns. Neat spermatozoa were characterized to demonstrate that coelenterazine and PBS neither contain nor cause the development of the observed round nanoscale structures in the CA-MNPs and Luc + MNP samples (Figs. 4 and 5).

## Abbreviations

BRET: bioluminescent resonance energy transfer-conjugated
MNPs: magnetic nanoparticles
CA: citric acid
CA-MNP: citric acid coated magnetic nanoparticles
Luc + MNP: core–shell structure with a luciferase shell around a CA-MNP core
BLI: bioluminescence imaging
QD: quantum dot
ART: assisted reproduction technologies
MRI: magnetic resonance imaging
TEM: transmission electron microscopy
PBS: phosphate buffer solution
Luc: luciferase
AFM/MFM: atomic and magnetic force microscopy
