# Supplementary material for: Bioluminescent magnetic nanoparticles as potential imaging agents for mammalian spermatozoa
Source: J Nanobiotechnology. 2016 Mar 17;14:20. doi: 10.1186/s12951-016-0168-y (PMC4794913; doi:10.1186/s12951-016-0168-y)
Supplement: Supplementary file 1 — 10.1186/s12951-016-0168-y Hydrodynamic diameter for in-house synthesized CA-MNPs, as measured by DLS number intensity (5 replicates). An average diameter of 31.5 ± 1.5 nm was measured for the in-house synthesized CA-MNPs; Figure S2. Hydrodynamic diameter for Luc + MNP, as measured by DLS number intensity (5 replicates). After luciferase addition, the number intensity particle diameter measured increased as compared to CA-MNPs. The larger particle diameter matches the size increases observed by TEM, and confirms that luciferase is bound onto the CA-MNP. Future studies will examine the optimum ratio between luciferase and CA-MNPs, the mechanisms of the adsorption process(es), and the resultant Luc + MNP complex structures. Figure S3. Magnetic nanoparticles (Luc + MNPs) aggregated under the presence of PBS solutions and in a conventional TEM characterization where like in the confocal, epifluorescence, and atomic/magnetic force microscopy results dried samples were used for imaging Luc + MNPs interacting with spermatozoa with the drying process contributing to the aggregation observed. Figure S4. QDs nanoparticles combined with spermatozoa showed binding at different sites on the cells (arrows) as well as unlabeled cells (arrow heads). Scale bar 10 micrometers. The QD associated with spermatozoa are attached at multiple and different sites on the cell; examples are marked with arrows. Note that not allspermatozoa were labeled using the QD method; examples of these unlabeled cells are marked with arrow heads. Figure S5. Neat spermatozoa observed with an epifluorescence microscope. Scale bar 10 microns. Neat spermatozoa were characterized to demonstrate that coelenterazine and PBS neither contain nor cause the development of the observed round nanoscale structures in the CA-MNPs and Luc + MNP samples (Figs. 4 and 5). [file 12951_2016_168_MOESM1_ESM.pdf]

Additional File

Bioluminescent Magnetic Nanoparticles as Potential Imaging Agents for Mammalian Spermatozoa

Erick S. Vasquez[1], *Jean M. Feugang*[2 3]*, *Scott T. Willard*[2 3 4], *Peter L. Ryan*[2 3 5], *Keisha B. Walters*[6]*

[1]Department of Chemical and Materials Engineering, University of Dayton, OH 45469

[2]Facility for Cellular and Organismal Imaging, Mississippi State University, MS 39762

[3]Department of Animal and Dairy Sciences, Mississippi State University, MS 39762

[4]Department of Biochemistry and Molecular Biology, Entomology and Plant Pathology, Mississippi State University, MS, 39762

[5]Department of Pathology and Population Medicine, College of Veterinary and Medicine, Mississippi State, MS, 39762

[6]Dave C. Swalm School of Chemical Engineering, Mississippi State University, MS 39762

Authors: evasquez1@udayton.edu, swillard@cals.msstate.edu, and ryan@provost.msstate.edu.
*Corresponding authors: jn181@ads.msstate.edu and kwalters@che.msstate.edu

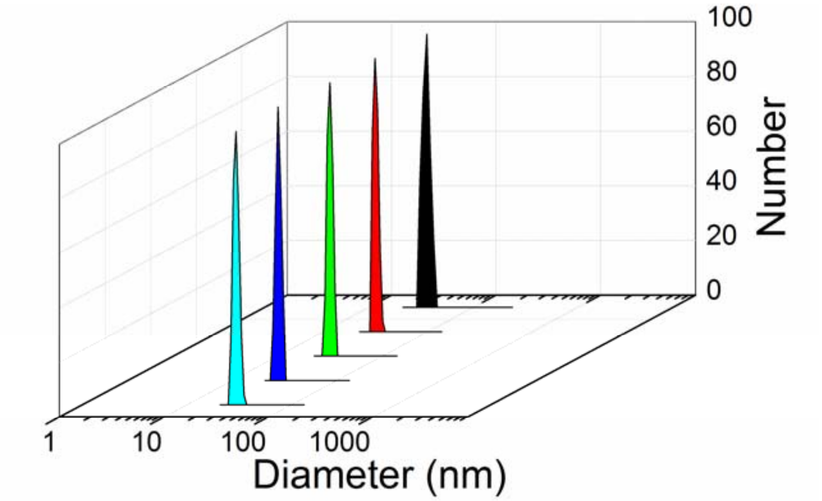

Figure S1. Hydrodynamic diameter for in-house synthesized CA-MNPs, as measured by DLS number intensity (5 replicates). An average diameter of 31.5 ± 1.5 nm was measured for the in-house synthesized CA-MNPs.

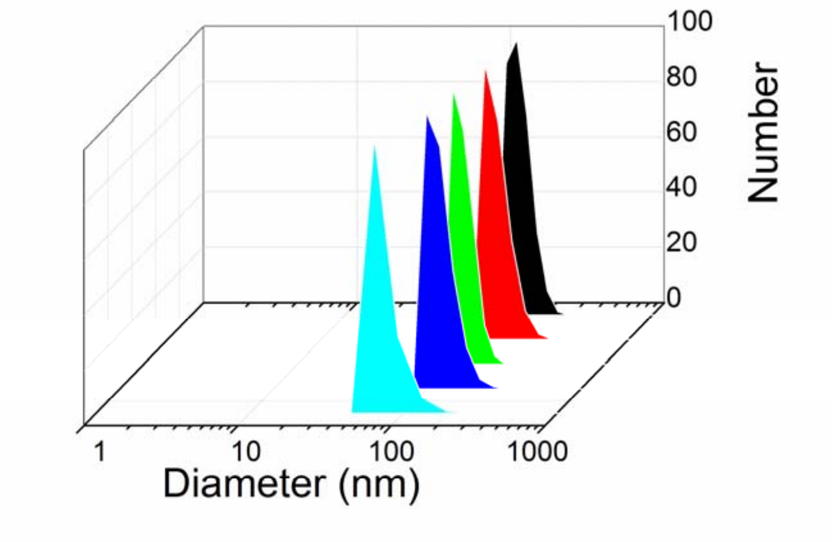

Figure S2. Hydrodynamic diameter for Luc+MNP, as measured by DLS number intensity (5 replicates). After luciferase addition, the number intensity particle diameter measured increased as compared to CA-MNPs. The larger particle diameter matches the size increases observed by TEM, and confirms that luciferase is bound onto the CA-MNP. Future studies will examine the optimum ratio between luciferase and CA-MNPs, the mechanisms of the adsorption process(es), and the resultant Luc+MNP complex structures.

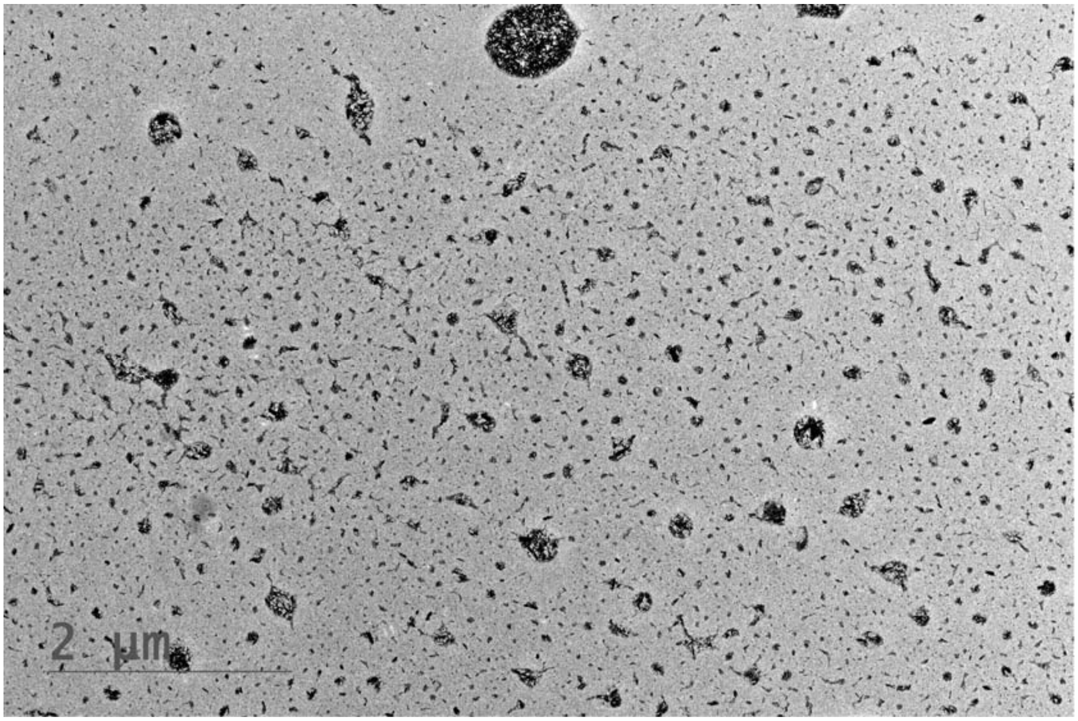

Figure S3. Magnetic nanoparticles (Luc+MNPs) aggregated under the presence of PBS solutions and in a conventional TEM characterization where like in the confocal, epifluorescence, and atomic/magnetic force microscopy results dried samples were used for imaging Luc+MNPs interacting with spermatozoa with the drying process contributing to the aggregation observed.

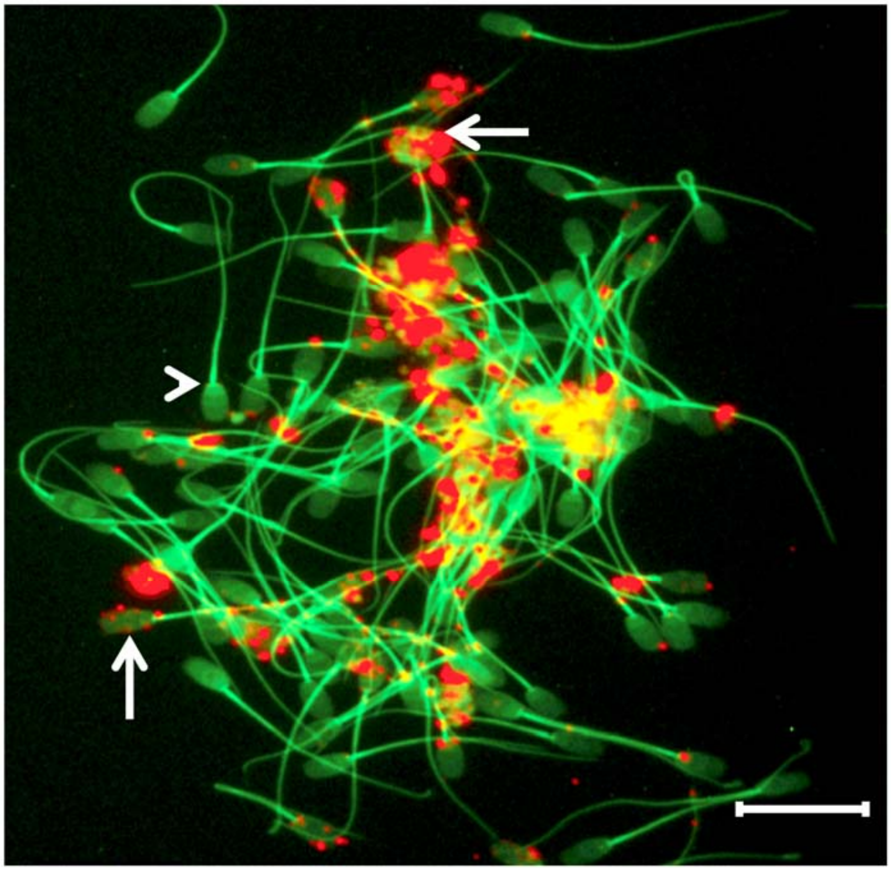

Figure S4. QDs nanoparticles combined with spermatozoa showed binding at different sites on the cells (arrows) as well as unlabeled cells (arrow heads). Scale bar = 10 micrometers. The QD associated with spermatozoa are attached at multiple and different sites on the cell; examples are marked with arrows. Note that not all spermatozoa were labeled using the QD method; examples of these unlabeled cells are marked with arrow heads.

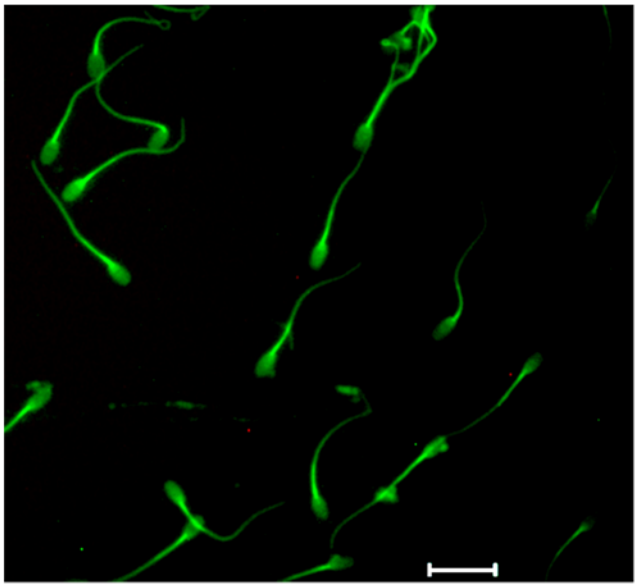

Figure S5. Neat spermatozoa observed with an epifluorescence microscope. Scale bar = 10 microns. Neat spermatozoa were characterized to demonstrate that coelenterazine and PBS neither contain nor cause the development of the observed round nanoscale structures in the CA-MNPs and Luc+MNP samples (Figs. 4 and 5).
